# Supplementary material for: HIVprotI: an integrated web based platform for prediction and design of HIV proteins inhibitors
Source: J Cheminform. 2018 Mar 9;10:12. doi: 10.1186/s13321-018-0266-y (PMC5845081; doi:10.1186/s13321-018-0266-y)
Supplement: Supplementary file 2 — Additional file 2. Source code of HIVProtI web server. [file 13321_2018_266_MOESM2_ESM.zip › HIVprotI_Source-code/hivprotI/weka-3-6-6/doc/weka/core/ContingencyTables.html]

ContingencyTables


---


|  |  |  |
| --- | --- | --- |
| **Overview** \| **Package** \| **Class** \| **Tree** \| **Deprecated** \| **Index** \| **Help** \| **Weka's home** | |  |
| **PREV CLASS**   **NEXT CLASS** | **FRAMES**    **NO FRAMES**     **All Classes** |  |
| SUMMARY: NESTED \| FIELD \| CONSTR \| METHOD | DETAIL: FIELD \| CONSTR \| METHOD |  |


---


## weka.core Class ContingencyTables

```
java.lang.Object
  weka.core.ContingencyTables
```

**All Implemented Interfaces:**: RevisionHandler

---

``` public class ContingencyTables extends java.lang.Object implements RevisionHandler ```

Class implementing some statistical routines for contingency tables.

**Version:**
:   $Revision: 1.8 $

**Author:**
:   Eibe Frank (eibe@cs.waikato.ac.nz)

---

**Constructor Summary**

`ContingencyTables()`


| **Method Summary** | |
| --- | --- |
| `static double` | `chiSquared(double[][] matrix, boolean yates)`             Returns chi-squared probability for a given matrix. |
| `static double` | `chiVal(double[][] matrix, boolean useYates)`             Computes chi-squared statistic for a contingency table. |
| `static boolean` | `cochransCriterion(double[][] matrix)`             Tests if Cochran's criterion is fullfilled for the given contingency table. |
| `static double` | `CramersV(double[][] matrix)`             Computes Cramer's V for a contingency table. |
| `static double` | `entropy(double[] array)`             Computes the entropy of the given array. |
| `static double` | `entropyConditionedOnColumns(double[][] matrix)`             Computes conditional entropy of the rows given the columns. |
| `static double` | `entropyConditionedOnRows(double[][] matrix)`             Computes conditional entropy of the columns given the rows. |
| `static double` | `entropyConditionedOnRows(double[][] train, double[][] test, double numClasses)`             Computes conditional entropy of the columns given the rows of the test matrix with respect to the train matrix. |
| `static double` | `entropyOverColumns(double[][] matrix)`             Computes the columns' entropy for the given contingency table. |
| `static double` | `entropyOverRows(double[][] matrix)`             Computes the rows' entropy for the given contingency table. |
| `static double` | `gainRatio(double[][] matrix)`             Computes gain ratio for contingency table (split on rows). |
| `java.lang.String` | `getRevision()`             Returns the revision string. |
| `static double` | `log2MultipleHypergeometric(double[][] matrix)`             Returns negative base 2 logarithm of multiple hypergeometric probability for a contingency table. |
| `static void` | `main(java.lang.String[] ops)`             Main method for testing this class. |
| `static double[][]` | `reduceMatrix(double[][] matrix)`             Reduces a matrix by deleting all zero rows and columns. |
| `static double` | `symmetricalUncertainty(double[][] matrix)`             Calculates the symmetrical uncertainty for base 2. |
| `static double` | `tauVal(double[][] matrix)`             Computes Goodman and Kruskal's tau-value for a contingency table. |

**Methods inherited from class java.lang.Object**

`equals, getClass, hashCode, notify, notifyAll, toString, wait, wait, wait`

**Constructor Detail**

### ContingencyTables

```
public ContingencyTables()
```


**Method Detail**

### chiSquared

```
public static double chiSquared(double[][] matrix,
                                boolean yates)
```

:   Returns chi-squared probability for a given matrix.

    :   **Parameters:**: `matrix` - the contigency table: `yates` - is Yates' correction to be used? **Returns:**: the chi-squared probability

---


### chiVal

```
public static double chiVal(double[][] matrix,
                            boolean useYates)
```

:   Computes chi-squared statistic for a contingency table.

    :   **Parameters:**: `matrix` - the contigency table: `useYates` - is Yates' correction to be used? **Returns:**: the value of the chi-squared statistic

---


### cochransCriterion

```
public static boolean cochransCriterion(double[][] matrix)
```

:   Tests if Cochran's criterion is fullfilled for the given
    contingency table. Rows and columns with all zeros are not considered
    relevant.

    :   **Parameters:**: `matrix` - the contigency table to be tested **Returns:**: true if contingency table is ok, false if not

---


### CramersV

```
public static double CramersV(double[][] matrix)
```

:   Computes Cramer's V for a contingency table.

    :   **Parameters:**: `matrix` - the contingency table **Returns:**: Cramer's V

---


### entropy

```
public static double entropy(double[] array)
```

:   Computes the entropy of the given array.

    :   **Parameters:**: `array` - the array **Returns:**: the entropy

---


### entropyConditionedOnColumns

```
public static double entropyConditionedOnColumns(double[][] matrix)
```

:   Computes conditional entropy of the rows given
    the columns.

    :   **Parameters:**: `matrix` - the contingency table **Returns:**: the conditional entropy of the rows given the columns

---


### entropyConditionedOnRows

```
public static double entropyConditionedOnRows(double[][] matrix)
```

:   Computes conditional entropy of the columns given
    the rows.

    :   **Parameters:**: `matrix` - the contingency table **Returns:**: the conditional entropy of the columns given the rows

---


### entropyConditionedOnRows

```
public static double entropyConditionedOnRows(double[][] train,
                                              double[][] test,
                                              double numClasses)
```

:   Computes conditional entropy of the columns given the rows
    of the test matrix with respect to the train matrix. Uses a
    Laplace prior. Does NOT normalize the entropy.

    :   **Parameters:**: `train` - the train matrix: `test` - the test matrix: `numClasses` - the number of symbols for Laplace **Returns:**: the entropy

---


### entropyOverRows

```
public static double entropyOverRows(double[][] matrix)
```

:   Computes the rows' entropy for the given contingency table.

    :   **Parameters:**: `matrix` - the contingency table **Returns:**: the rows' entropy

---


### entropyOverColumns

```
public static double entropyOverColumns(double[][] matrix)
```

:   Computes the columns' entropy for the given contingency table.

    :   **Parameters:**: `matrix` - the contingency table **Returns:**: the columns' entropy

---


### gainRatio

```
public static double gainRatio(double[][] matrix)
```

:   Computes gain ratio for contingency table (split on rows).
    Returns Double.MAX\_VALUE if the split entropy is 0.

    :   **Parameters:**: `matrix` - the contingency table **Returns:**: the gain ratio

---


### log2MultipleHypergeometric

```
public static double log2MultipleHypergeometric(double[][] matrix)
```

:   Returns negative base 2 logarithm of multiple hypergeometric
    probability for a contingency table.

    :   **Parameters:**: `matrix` - the contingency table **Returns:**: the log of the hypergeometric probability of the contingency table

---


### reduceMatrix

```
public static double[][] reduceMatrix(double[][] matrix)
```

:   Reduces a matrix by deleting all zero rows and columns.

    :   **Parameters:**: `matrix` - the matrix to be reduced **Returns:**: the matrix with all zero rows and columns deleted

---


### symmetricalUncertainty

```
public static double symmetricalUncertainty(double[][] matrix)
```

:   Calculates the symmetrical uncertainty for base 2.

    :   **Parameters:**: `matrix` - the contingency table **Returns:**: the calculated symmetrical uncertainty

---


### tauVal

```
public static double tauVal(double[][] matrix)
```

:   Computes Goodman and Kruskal's tau-value for a contingency table.

    :   **Parameters:**: `matrix` - the contingency table **Returns:**: Goodman and Kruskal's tau-value

---


### getRevision

```
public java.lang.String getRevision()
```

:   Returns the revision string.

    :   **Specified by:**: `getRevision` in interface `RevisionHandler`
    :   **Returns:**: the revision

---


### main

```
public static void main(java.lang.String[] ops)
```

:   Main method for testing this class.


---


|  |  |  |
| --- | --- | --- |
| **Overview** \| **Package** \| **Class** \| **Tree** \| **Deprecated** \| **Index** \| **Help** \| **Weka's home** | |  |
| **PREV CLASS**   **NEXT CLASS** | **FRAMES**    **NO FRAMES**     **All Classes** |  |
| SUMMARY: NESTED \| FIELD \| CONSTR \| METHOD | DETAIL: FIELD \| CONSTR \| METHOD |  |


---
